# Modular de- and re-construction of vascularized osteochondral tissues in an Organ-on-Chip dual-compartment platform

**DOI:** 10.1016/j.jot.2025.10.009

**Published:** 2025-11-27

**Authors:** Andrea Mainardi, Andrea Barbero, Martin Ehrbar, Marco Rasponi, Ivan Martin, Paola Occhetta

**Affiliations:** a Department of Biomedicine, University Hospital Basel, University of Basel, Hebelstrasse 20, 4031, Basel, Switzerland; b Department of Obstetrics, University Hospital Zurich, Frauenklinikstrasse 10, 8091, Zurich, Switzerland; c Zurich Centre for Integrative Human Physiology, Winterthurerstrasse 190, 8057, Zürich, Switzerland; d Department of Electronics, Information and Bioengineering, Politecnico di Milano, Via Golgi 39, 20133, Milan, Italy; e BiomimX S.r.l., Viale Decumano 41, 20157, Milan, Italy

**Keywords:** Organs-on-chip, In vitro model, Osteoarthritis, Bone-cartilage interface, Vascularization, Bottom-up approach

## Abstract

**Background:**

Homeostasis at the cartilage–bone interface of articular joints depends on tightly orchestrated signalling among chondrocytes, osteogenic progenitors, and subchondral vasculature. Disruption of this crosstalk is considered one of the main drivers of osteoarthritis (OA), the most prevalent musculoskeletal disease worldwide. However, the timing, location, and mechanisms underlying the pathological onset of OA remain unclear, hindering the development of targeted regenerative strategies. This knowledge gap emphasises the need for *in vitro* models that replicate OA's multi-tissue crosstalk in a representative yet accessible format.

**Methods and results:**

Here, we present a modular, dual-compartment Organ-on-Chip (OoC) platform that enables the stepwise ‘de- and re-construction’ of the vascularized osteochondral unit, allowing systematic interrogation of cell-specific roles in homeostasis and inflammation. Through the side-by-side culture of human articular chondrocytes (hACs) and bone marrow-derived mesenchymal stromal cells (bmMSCs), we generated biphasic, compartmentalized constructs with a contiguous interface, in which bmMSCs exhibited osteogenic commitment without compromising the stable chondrogenic capacity of hACs. The addition of human umbilical vein endothelial cells (HUVECs) to the bmMSCs compartment at a finely tuned 3:2 ratio (bmMSCs:HUVECs) enabled the formation of lumenized vascular vessels surrounded by α-SMA–expressing cells and laminin sheaths, while preserving bmMSCs' osteogenic commitment. Under homeostatic conditions, the presence of a cartilage layer adjacent to such vascularized and mineralized tissue impeded vascular and stromal invasion, whereas exposure to IL-1β (1 ng/mL) allowed to override such chondrocyte “barrier,” triggering endothelial and stromal penetration into the cartilage, thus mimicking inflammatory OA.

**Conclusion:**

The proposed platform combines ease of use, real-time imaging capabilities, and precise control over cellular modules, offering a versatile tool for future mechanistic studies in OA and related joint disorders.

**The translational potential of this article:**

This modular Organ-on-Chip platform offers a physiologically relevant and experimentally accessible model of the vascularized osteochondral interface, enabling systematic dissection of cell-specific roles in joint homeostasis and inflammation. By recapitulating key features of early osteoarthritic pathology—including barrier breakdown, stromal invasion, and endothelial remodeling— with a highly modular and technologically robust approach, this system holds translational promise for preclinical testing of disease-modifying OA therapies, biomarker discovery, and regenerative strategies targeting cartilage–bone crosstalk.

## Graphical abstract

**Figure:**
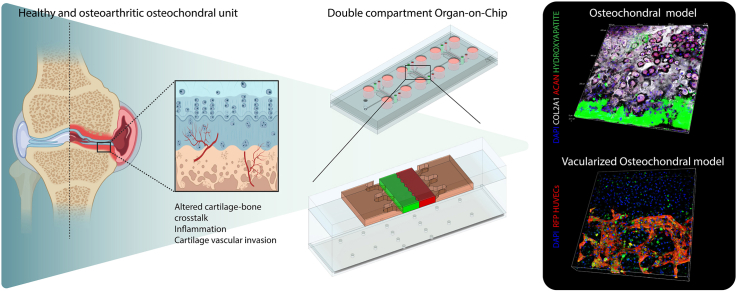

## Introduction

In diarthrodial joints, the cartilage–bone interface—comprising articular cartilage, calcified cartilage, and the subchondral bone plate—plays a crucial role in load transmission during weight-bearing and gait [1,2]. At this highly integrated interface, communication among chondrocytes, osteogenic progenitors, and microvessels is tightly regulated by biochemical and mechanical cues [1,3]. Disruption of this multicellular crosstalk has been identified as a key driver of osteoarthritis (OA), the most prevalent musculoskeletal disease worldwide. However, the timing, causal relationships, and the specific tissue within the cartilage–bone interface where these changes are initiated remain debated [4,5]. This knowledge gap hampers the development of effective regenerative therapies that directly target OA pathological triggers and underscores the need for *in vitro* models that capture the interplay among multiple joint tissues, helping to unravel OA mechanisms and accelerate therapy discovery.

In this framework, several studies aimed at replicating the biphasic cartilage–bone architecture using either stacked 3D-printed constructs [[6], [7], [8]] or scaffolds integrating patterned structures [9,10]. While these approaches provide high spatial fidelity and tissue-relevant dimensions, their millimetre-scale thicknesses—orders of magnitude larger than the cellular length scale—limit their suitability for studying cell-driven phenomena, largely due to challenges in precisely controlling microenvironmental cues in such volumes.

Miniaturized three-dimensional systems—i.e., Organ-on-Chip (OoC) platforms—offer a promising approach to model the onset and progression of OA by reconstructing the microenvironment of the cartilage-to-bone interface at a cellular relevant scale [11]. Existing joint-on-chip models have demonstrated the ability to reproduce the dynamic biochemical and biomechanical cues present in OA joints, provide spatially controlled 3D environments, and support the co-culture of multiple joint tissues and cell types [[12], [13], [14], [15], [16], [17], [18], [19], [20], [21]].

Focusing on the osteochondral compartment, pioneering OoC-based models have employed bone marrow derived mesenchymal stromal cells (bmMSCs) as a single cell source to reconstruct both cartilage and bone tissues and to promote the formation of their interface. To this aim, the direct differentiation of bmMSCs toward both osteogenic and chondrogenic lineages was pursued inside a single micropatterned hydrogel [16,22]. However, this approach requires long culture periods to achieve sufficient cellular differentiation and overlooks bmMSCs’ intrinsic commitment to follow the endochondral ossification pathway rather than generate stable articular cartilage [23].

For this reason, alternative strategies have been introduced to develop biphasic osteochondral microtissues composed of distinct cell sources arranged in adjacent phases. These models include osteochondral constructs formed by human articular chondrocytes (hACs) and osteoblasts co-cultured within side-by-sides hydrogels that induce the formation of tissue-specific oxygen gradients [24], setups separated by physical membranes [20], or tissues populated with gender-specific cells to assess sex-dependent inflammatory responses [25]. However, terminally differentiated osteoblasts are unable to recapitulate the dynamic endochondral ossification processes that establish the native osteochondral interface during development and are reactivated during OA progression [26]. In contrast, bmMSCs provide a more physiologically relevant cell source, as they inherently undergo endochondral ossification and can form tissues mimicking the mineralized cartilage transition zone — a functional hallmark of the osteochondral unit [27].

An equally critical yet often underexplored aspect of the cartilage–bone interface in the aforementioned models is the incorporation of a functional vascular component. Enhanced angiogenesis in the subchondral bone and ectopic invasion of microvessels into the cartilage are well-recognized clinical hallmarks of OA progression, correlating with matrix calcification, sensory nerve ingrowth, and pain [28]. Thus, faithfully recreating the dynamic angiogenic front at the cartilage–bone interface—and allowing direct visualisation of its invasion into the cartilage layer—is essential for capturing disease-relevant phenotypes.

To this end, a few recently introduced microscale models aimed to mimic the multicellular environment of the vascularized cartilage–bone interface through the co-culture of up to five cell types within the same platform (i.e., human umbilical vein endothelial cells (HUVECs), bmMSCs, osteoblasts, hACs, and macrophages) (e.g. Refs. [21,29]). Although these multicellular models have achieved an unprecedented degree of complexity, their biological intricacy makes it difficult to isolate and interrogate the specific contributions of each cell population to OA-related phenotypic changes. Furthermore, these platforms typically rely on technologically sophisticated multilayer devices necessitating manual, user-dependent assembly (e.g. Ref. [21]), a process that limits throughput, compromises robustness, and constrains their practicality as *in vitro* tools for the systematic study of cell-specific roles.

To overcome these limitations, here, we propose a stepwise “de- and re-construction” strategy: by first isolating the essential joint-interface modules—namely vascular, cartilaginous, and mineralized tissues—and then recombining them into spatially controlled modular configurations, we can precisely interrogate how each cell type drives physiological development or pathological changes at the osteochondral boundary. Specifically, we present a bottom-up OoC platform that allows the assembly of three well-defined cellular components—hACs, bmMSCs, and HUVECs—within an easy-to-operate dual-compartment device, providing direct optical access to the forming microtissue interface. This approach enables the systematic engineering of a vascularized osteochondral interface, which permits to (i) evaluate hACs’ and bmMSCs’ phenotypic changes when cultured alone, in combination, or alongside HUVECs; (ii) directly monitor cartilage–bone crosstalk and inflammation-driven vascular invasion; and (iii) maintain the simplicity required for reproducible, high-content OA studies.

## Materials and methods

### Dual-compartment microscale device design

The dual-compartment microscale device was designed as follows. The platform comprises three identical chambers (i.e. functional units), so that each platform contains a triplicate of independently cultured microconstructs. Each functional unit consists of two central compartments (each 300 μm wide) to host the cell-laden hydrogels, separated by a row of hexagonal pillars (50 μm wide, 90 μm long, with a 50 μm spacing between adjacent pillars). The hexagonal shape of the pillars was chosen to prevent leakage of the cell-laden hydrogels upon injection while maximizing the interface area between the two constructs. The two central compartments are flanked on their outer surfaces by T-shaped pillars, spaced 30 μm apart, with arms 300 μm long and 100 μm wide. This design replicates the geometry of the external pillars of a device that we previously published [15](uBeat® Compress Platform, BiomimX®), and which was used for single cultures in this study. Adjacent to the T-shaped pillars are the culture medium channels, with a width of 765 μm from the outer edge of the hydrogel. The total height of the chamber is 143 μm, while the height of both the hexagonal and T-shaped pillars is 100 μm. The bottom of the culture chamber is formed by a smooth, 800 μm-thick membrane positioned over an actuation chamber, enabling future application of mechanical stimuli, though such stimulation was not implemented in this study.

### Microfluidic devices fabrication

Devices were fabricated using soft lithography techniques: master moulds were created via photolithography; polydimethylsiloxane (PDMS) platforms were produced by replica moulding.

Full-scale device drawings were created using Computer-Aided Design (CAD) software (AutoCAD 2024, Autodesk), converted to.gds files (LayoutEditor), and translated into physical structures through multilayer direct laser writing (Heidelberg MLA100) of SU-8 2035 or SU-8 2100 photoresists (MicroChem) onto 102 mm silicon wafer substrates. All operations were performed in a cleanroom environment (Class ISO6). Master moulds were subsequently exposed to trimethylchlorosilane (TMCS, Sigma–Aldrich) vapour at room temperature for 30 min to facilitate demoulding.

PDMS devices were fabricated by mixing base and curing agent at a 10:1 wt ratio, as specified by the manufacturer (Sylgard 184, Dow Corning). The mixture was degassed in a vacuum chamber at −0.8 bar for 15 min to remove air bubbles, poured onto the appropriate moulds, and subjected to a second degassing cycle under the same conditions. The polymer was then cured on a levelled shelf at 65 °C for 2.5 h. Subsequently, the PDMS layers were carefully peeled from the moulds and trimmed with a razor blade. Holes for the culture medium reservoirs (5 mm diameter), gel channel inlets (1 mm diameter), and actuation chamber (1.5 mm diameter) were created using biopsy punchers. Layers were bonded via air plasma treatment (Harrick Plasma) and brought into conformal contact for at least 30 min at 80 °C to achieve irreversible bonding. Finally, devices were sterilized by autoclaving and further cured at 70 °C for 24 h to minimize residual leachates from uncured PDMS. [30]. Devices with a single hydrogel chamber were purchased from the manufacturer (uBeat® Compress30 Platform, BiomimX® Srl).

### PEG hydrogels preparation

PEG hydrogels were produced as previously described [31]. 1 ml of FXIII (200 U ml^−1^; Fibrogammin; CSL Behring) was activated with 100 μl of thrombin (20 U ml^−1^; Sigma–Aldrich) for 30 min at 37 °C and the resulting activated FXIII (FXIIIa) was stored in small aliquots at −80 °C. Eight-arm PEG vinylsulfone (molecular weight: 40 kDa; NOF Europe) was functionalized with peptides that contained either an FXIII glutamine acceptor substrate (Gln peptides; NQEQVSPL-ERCG-NH2; Bachem) or an MMP-degradable FXIII lysine donor substrate (Lys-MMPsensitive peptides; Ac-FKGGGPQGIWGQ-ERCG-NH2; Bachem), resulting in 8-PEG-Gln or 8-PEG-MMPsensitive-Lys precursors, respectively. A stoichiometrically balanced solution of 8-PEG-Gln and 8-PEG-MMP sensitive-Lys for the indicated final dry mass content of hydrogel precursors was mixed in Tris buffer (50 mM Tris (pH 7.6)) containing 50 mM calcium chloride, leaving a spare volume of 10 % v/v for the addition of culture medium and cells. Hydrogel cross-linking was initiated by adding 10 U ml^−1^ FXIIIa and vigorous mixing. Hydrogels adopted in the study had a final concentration of 2 % PEG precursor.

### hACs procurement and expansion

Primary hACs were harvested from the knee joint cartilage of 7 individuals (age 54 ± 15 years, 4 males, 3 females, Table S1) with no clinical history of OA or other degenerative diseases, and without signs of cartilage surface fibrillation in the harvested area (i.e. from healthy cartilage tissues). Tissue harvesting was performed during knee surgery due to traumatic injuries or from cadaver donors during autopsy procedures. Harvesting processes were performed under the general hospital consent (University Hospital of Basel, Switzerland), following informed consent from patients or relatives, and in accordance with the Ethical Committee of Northwest and Central Switzerland (EKNZ). When applicable, samples were collected under ethical approval EKNZ 2014-199.

Cartilage samples were minced with a scalpel, enzymatically digested with a solution of 0.15 % (w/v) type II collagenase (300 U mg^−1^; Worthington Biochemical Corporation) - 10 ml of solution per g of tissue, 37 °C, 22 h - and resuspended in Dulbecco’s modified Eagle’s medium (DMEM) containing, 4.5 mg ml^−1^ d-glucose, 0.1 mM non-essential amino acids, 1 mM sodium pyruvate, 100 mM HEPES buffer, 100 U ml^−1^ penicillin, 100 μg ml^−1^ streptomycin, 0.29 mg ml^−1^ l-glutamine, and 10 % foetal bovine serum (FBS), i.e. complete medium. Isolated hACs were counted using Trypan blue (Thermo-Fisher), plated in culture flasks at a density of 10^4^ cells cm^−2^, and expanded (in a humidified incubator, 37 °C, 5 % CO_2_) in complete medium supplemented with 1 ng ml^−1^ of transforming growth factor-β1 (TGF-β1) and 5 ng ml^−1^ of fibroblast growth factor-2 (FGF-2). hACs were expanded up to 80 % confluency, then detached from flasks by incubation with a solution of 0.05 % trypsin/0.53 mM EDTA (Gibco) for 5 min at 37 °C. Cells were either immediately replated using the same medium and initial seeding density, or frozen and stored in liquid nitrogen. Freezing was performed using a freezing medium (FM) consisting of 10 % (v/v) dimethyl sulfoxide (DMSO, D2650, Hybri-Max™, Sigma–Aldrich) in FBS. hACs were suspended in FM (1.6 × 10^6^ cells ml^−1^), aliquoted into cryogenic preservation vials (Sarstedt), and slow frozen using a Mr. Frosty™ Freezing Container cooled to −80 °C. After 2 days at −80 °C, vials were transferred to a liquid nitrogen–based dewar for long-term cryogenic storage. Thawing was performed by placing the cryogenic preservation vials in a 37 °C water bath for 1 min, followed by centrifugation (3 min, 700 RCF) and washing with complete medium. After each freeze/thaw cycle, cell viability (consistently above 90 %) was assessed using a Luna Automatic Cell Counter (Logos Biosystems) with Trypan Blue staining (0.4 % v/v, Gibco). hACs at passage 2–3 were used in experiments. After expansion, hACs were detached using a solution of 0.05 % trypsin/0.53 mM EDTA (Gibco), counted, and injected into OoC devices.

### bmMSCs procurement and expansion

Primary human bmMSCs were isolated from bone marrow aspirates (20 mL volume) from 5 healthy donors (age: 34 ± 13 years, 3 males, 2 females, Table S1) during routine orthopaedic procedures involving exposure of the iliac crest. Harvesting procedures were performed after informed consent and in accordance with the local ethics committee (University Hospital Basel, Switzerland). A bone-marrow biopsy needle (Argon medical devices) was inserted through the cortical bone and the aspirate was immediately transferred into plastic tubes containing 15,000 IU of heparin. Isolated bmMSCs were counted using Crystal violet, plated in tissue culture flasks, and expanded in a humidified incubator (37 °C, 5 % CO_2_) in minimum essential medium eagle - Alpha modification (α-MEM), containing 10 % FBS, 4.5 mg ml^−1^ D-glucose, 0.1 mM nonessential amino acids, 1 mM sodium pyruvate, 100 mM Hepes buffer, 100 Ul ml^−1^ penicillin, 100 μg ml^−1^ streptomycin, and 0.29 mg ml^−1^ L-glutamate, and further supplemented with 5 ng ml^−1^ of FGF-2 (R&D Systems). The medium was changed twice a week. After approximately 10 days, when they were about 80 % confluent, cells were rinsed with phosphate buffered saline (PBS), detached using 0.05 % trypsin/0.53 mM EDTA (Gibco), and replated at 5 × 10^3^ cells cm^−2^. Passage 3–4 bmMSCs were used in on-chip experiments. Expansion, freezing, and thawing were performed as described for hACs.

### HUVECs procurement and expansion

Red fluorescent protein (RFP)-positive human umbilical vein endothelial cells (HUVECs) were purchased from Angio-Proteomie (Boston, MA, USA). Cells were seeded at a density of 6 × 10^3^ cells cm^−2^ and expanded as a monolayer using Endothelial Growth Medium 2 (EGM-2; Lonza, Basel, Switzerland) in cell culture flasks coated with a 1 % (w/v) gelatine solution (Sigma–Aldrich) in PBS (Gibco). Passaging was performed at 80 % confluency by detaching cells with 0.05 % trypsin/0.53 mM EDTA (Gibco), as described above. Freezing and thawing were performed as described for hACs. Cells at passage 4 were used in on-chip experiments.

### Cartilaginous, mineralized, and double constructs injection and maturation in OoC devices

hACs and bmMSCs constructs were prepared by embedding the cells in the enzymatically crosslinkable and MMP-sensitive PEG hydrogel formulation described above [3]. Cells were detached from flasks, counted using trypan blue (Gibco), and mixed with the polymer precursor to achieve a final cell density of 50 × 10^6^ cells ml^−1^ of hydrogel. The cell–polymer precursor solution was then mixed with 10 U ml^−1^ thrombin-activated factor FXIIIa and immediately injected into microscale devices. Complete crosslinking of the hydrogel was achieved by incubating the constructs for 10 min at 37 °C in a 5 % CO_2_ atmosphere. This procedure was repeated for the second channel when needed for double constructs. Upon complete polymerization of the hydrogel, the lateral medium channels and reservoirs were filled with the appropriate differentiation medium.

Chondrogenic medium (CHM) consisted of DMEM (Sigma–Aldrich) supplemented with 4.5 mg ml^−1^ D-glucose, 0.1 mM non-essential amino acids, 1 mM sodium pyruvate, 100 mM HEPES buffer, 100 U ml^−1^ penicillin, 100 μg ml^−1^ streptomycin, 0.29 mg ml^−1^ L-glutamine, 1.0 mg ml^−1^ insulin, 0.55 mg ml^−1^ human transferrin, 0.5 μg ml^−1^ sodium selenite, 50 mg/ml bovine serum albumin, 470 μg ml^−1^ linoleic acid, and 1.25 % human serum albumin, 0.1 mM ascorbic acid 2-phosphate, 10^−4^ mM dexamethasone, and 10 ng/ml TGF-β3. Osteochondral medium (OCM) was prepared by adding 10 mM β-glycerophosphate (β-Gly, Sigma–Aldrich) to the CHM. The culture medium was changed every two days; samples were maintained under static conditions for 14 days.

### Vascular constructs maturation

Vascular constructs were obtained mixing bmMSCs and HUVECs with bmMSCs: HUVECs rations of 1:10, 1:2, and 3:2 in the above mentioned enzymatically crosslinkable and MMP-sensitive PEG hydrogel [3]. Preliminary experiments were performed considering both a total cellular density (i.e. obtained considering the sum of bmMSCs and HUVECs) of 50 × 10^6^ cells ml^−1^, i.e. the same density used for hACs and bmMSCs constructs described above, and of 10 × 10^6^ cells ml^−1^ as previously published in other co-culture works [32]. Cells were injected into OoC devices as described above and cultured for either 7 or 14 days in static conditions, using a 1:1 mixture of OCM and EGM-2 but compensating so that the final concentration of EGM-2 growth factors would not be diluted by a factor of 2. Medium was changed every other day. Tissues constituted by only bmMSCs and exposed either to the OCM: EGM-2 mixture or to simple OCM were used as controls for the maturation of vascular constructs.

### Creation of vascularized osteochondral constructs

Vascularized osteochondral constructs were obtained using double compartment devices. One of the channels was injected with a 3:2 mixture of bmMSCs and HUVECs at a total density of 50 × 10^6^ cells ml^−1^; the second one was injected with either (GFP^+^) hACs at a density of 50 × 10^6^ cells ml^−1^ or with empty PEG hydrogels used as controls. Constructs were cultured in the OCM: EGM-2 mixture, for 7 days, in static conditions. To assess the capacity of hACs constructs to resist vascular invasion, the experiment was also repeated with and without the supplementation of 1 ng ml^−1^ of the pro inflammatory cytokine Interleukin-1 beta (IL-1β), which was added to the culture medium from day 3 to day 7. Controls were supplemented with equal volumes of DMSO (Sigma Aldrich) which was used as a vehicle for IL-1β.

### Cell transduction

GFP^+^ hACs were obtained via lentiviral transduction, using a previously published protocol shown not to affect the chondrogenic differentiation capacity of the cells [33]. Briefly, hACs were seeded at a density of 10^4^ cells cm^−2^ and transduced the following day with 2.8 × 10^6^ transducing units (TU) of GFP lentivirus at a multiplicity of infection (MOI) of 5, in the presence of 5 μg ml^−1^ protamine sulfate. Three days post-transduction, cells were detached and GFP^+^ cells were isolated by fluorescence-activated cell sorting (FACS) using a BD FACSAria III cell sorter. GFP^+^ hACs were subsequently expanded and used at passages 3–5.

mCherry^+^ bmMSCs were similarly obtained via lentiviral transduction. Lenti-X 293T cells (Clontech, USA) were transfected with the lentiviral expression vector pLVX_mCherry (Clontech backbone) along with third-generation packaging plasmids prMDLg/pREE, pRSV-Rev, and pMD2.G (Addgene #12251, #12253, and #12259, respectively) using Lipofectamine 2000 (Thermo Fisher, #11668019). After 72 h, the supernatant containing the lentiviral particles was collected, and the viral titer was determined by ELISA using the QuickTiter Lentivirus Titer Kit (Cell Biolabs, #VPK-1070). For transduction, bmMSCs were seeded at a density of 10^4^ cells cm^−2^, and lentiviral particles were added the following day at an MOI of 1 in the presence of 8 μg ml^−1^ polybrene (Sigma, #107689). Viral particles were removed after 12 h. Starting from 48 h post-transduction, bmMSCs were selected with 8 days of G418 (Geneticin, Roche, #4727878001) treatment. mCherry^+^ bmMSCs were then expanded and used at passages 4–5.

### Gene expression analysis

Gene expression was quantified through quantitative reverse transcription polymerase chain reaction (RT-qPCR). At the end of the culture period, cellular constructs were harvested by peeling off the bottom PDMS layers of the device, carefully scraping the constructs with a pipette tip, and transferring them into an Eppendorf tube containing 400 μL of TRI Reagent® (T9424, Sigma–Aldrich). Total RNA was extracted using TRI reagent ® (T9424, Sigma Aldrich), according to the manufacturer instructions. Glycerol (G5516, Sigma–Aldrich) was adopted to enhance RNA precipitation. Reverse transcription and complementary DNA (cDNA) synthesis were performed using the SuperScript III Reverse Transcriptase Kit (18080093, Invitrogen). RT-qPCR was performed according to standard protocols, using the Applied Biosystems 7300 Real-Time PCR system and equalising the amount of cDNA present in each reaction well. The following gene of interests were quantified using TaqMan probes (Thermo Fisher): *COL2A1* (Hs00264051_m1), *COL1A1* (Hs00164004_m1), *ACAN* (Hs00153936_m1), *COL10A1* (Hs00166657_m1), *IHH* (Hs01081800_m1), *BSP2* (Hs00173720_m1), *ALP* (Hs01029144_m1), *OCN* (Hs01587814_g1), *OPN* (Hs00959010_m1). *GAPDH* (Hs02758991_g1) was used as housekeeping gene.

### Immunofluorescence analyses

Immunofluorescence analyses were performed directly within OoC devices. At the end of the culture period, samples were washed with PBS and fixed overnight at 4 °C with a 4 % (w/v) paraformaldehyde solution. Devices were subsequently disassembled by peeling off the actuation membrane to expose the cellular constructs. Cells were permeabilized with a solution of 0.5 % (v/v) Triton X-100 (Sigma–Aldrich) in PBS; non-specific binding was blocked incubating samples for 1 h at room temperature with a solution containing 0.3 % (v/v) Tween-20 and 3 % (v/v) goat serum in PBS.

Samples were then incubated overnight at 4 °C with primary antibodies. The following antibodies were used to assess the maturation of cartilaginous and mineralized constructs: aggrecan (ACAN, Abcam, ab36861, dilution 1:200), collagen type II (COL2A1, Abcam, ab185430, 1:200), osteocalcin (OCN, Millipore, ab1857, 1:200), and alkaline phosphatase (ALP, Abcam, ab54778, 1:200). To evaluate cell heterogeneity in hACs–bmMSCs co-cultures, we used osteopontin (OPN, Proteintech, 22952-1, 1:200), platelet-derived growth factor receptor beta (PDGFRB, R&D Systems, AF1042, 1:200), and nestin (Millipore, MAB5326, 1:200). Bone sialoprotein (BSP, Abcam, ab52128, 1:200), laminin (Abcam, ab11575, 1:200), and alpha-smooth muscle actin (α-SMA, Sigma–Aldrich, A2547, 1:400) were used to characterise the phenotype of the vascular constructs.

Samples were washed twice with blocking solution (20 min each at room temperature) following primary antibody incubation, then incubated for 1 h at room temperature with secondary antibodies conjugated to Alexa Fluor 488, 546, or 647 (Invitrogen, 1:200), and subsequently washed with PBS. Nuclear staining was performed using 4′,6-diamidino-2-phenylindole (DAPI). Deposition of hydroxyapatite (HA) was assessed using the OsteoImage mineralisation assay (Lonza), following the manufacturer’s instructions. GFP^+^ hACs, mCherry^+^ bmMSCs, and RFP^+^ HUVECs were imaged directly, without additional staining.

Immunofluorescence imaging was performed using a Nikon AxR confocal microscope and a Nikon Crest V3 spinning disk microscope. Brightfield images acquired during the culture period were captured using a brightfield microscope with an integrated camera (EVOS XL Core, Life Technologies). Image analysis was conducted using ImageJ and QuPath software.

### Statistical analysis

RT-qPCR results are presented as mean +standard deviation. Image quantifications analyses are presented as mean ± standard deviation. Single data points were plotted to account for non-normal distributions. Populations’ normality was verified using Shapiro–Wilk and Kolmogorov–Smirnov tests unless otherwise specified. Non-Paired double comparisons were performed using two tailed t-test for normal populations and Mann–Whitney test for non-Gaussian populations. Multiple comparisons were performed using ordinary one-way ANOVA with Tukey’s multiple comparison tests for normal distributions and Kruskal–Wallis test with Dunn’s multiple comparison tests for non-normal distributions.

## Results

### Definition of a medium composition driving chondrogenic differentiation of hACs and osteogenic differentiation of bmMSCs

Firstly, we assessed the effect of a source of inorganic phosphate (i.e. beta-glycerophosphate, β-Gly) in modulating hACs’ and bmMSCs’ chondrogenic differentiation. To this aim, hACs or bmMSCs isolated from healthy donors were embedded in an enzymatically crosslinked and MMP-sensitive PEG-based hydrogel [31] and injected into a previously developed single chamber OoC device [15] (uBeat® Compress30 Platform, BiomimX® S.r.l.). Cells were cultured for 14 days in a serum free medium formulation demonstrated to favour chondrogenic differentiation [34] – i.e. chondrogenic medium (CHM) - or in an osteochondral culture medium formulation (OCM), consisting of CHM further supplemented with β-Gly.

**Fig. 1:**
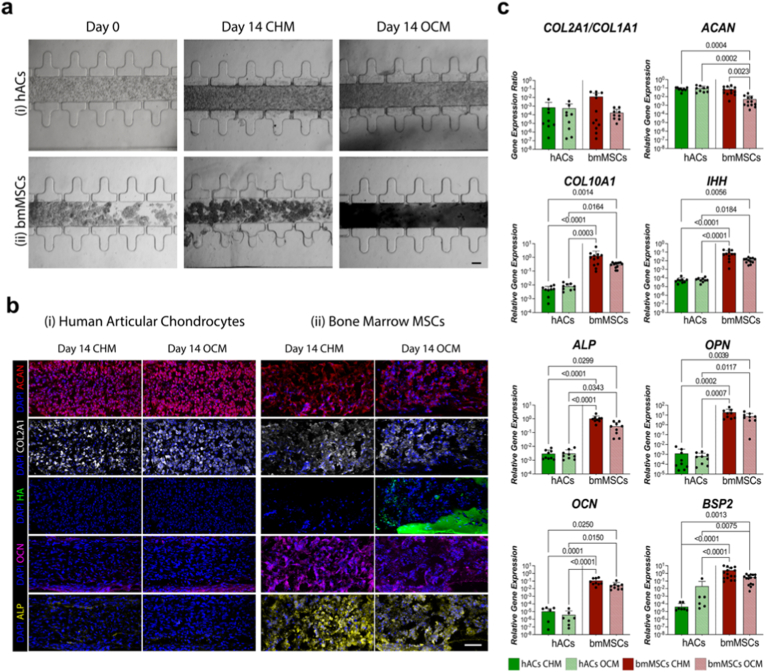
**OCM drives hACs chondrogenesis and bmMSCs hypertrophic/osteogenic differentiation. a.** Brightfield pictures of hACs (i) and bmMSCs (ii) constructs at day 0 and after 14 days of differentiation in CHM or OCM. hACs and bmMSCs were cultured for 14 days in a previously published Cartilage-on-Chip (CoC) device [15]. bmMSCs construct appeared black upon exposure to OCM, consistently with the deposition of calcium salts (n ≥ 9 independently cultured samples from n ≥ 5 donors for each cell type). Scale bar, 100 μm. **b.** Immunofluorescence images of hACs (i) and bmMSCs (ii) constructs cultured in CHM or OCM for 14 days (n ≥ 3 independently cultured samples from n ≥ 3 donors for each condition). Scale bar, 100 μm. **c.** RT-qPCR quantification of hACs and bmMSCs gene expression upon 14 days of differentiation in CHM or OCM (n ≥ 9 independently cultured samples from n ≥ 3 donors). Statistics by Kruskal–Wallis test with Dunn’s post hoc test for multiple comparisons. Statistical significance was assessed both between the same cell type (i.e. upon CHM or OCM conditioning) and between hACs and bmMSCs. Gene expression was normalized to GAPDH expression. Values are reported as mean +s.d. Populations normality was tested using Shapiro–Wilk and Kolmogorov–Smirnov tests. (Adjusted) P values < 0.05 are reported on the graph.

After 14 days in culture, tissues generated by culturing hACs in both CHM and OCM exhibited a dense matrix but showed no evidence of dark calcium deposits (Fig. 1a(i)). Immunofluorescence analyses confirmed that, irrespective of the medium formulation used, hACs formed stable cartilage constructs rich in collagen type II (COL2A1) and aggrecan (ACAN), while staining negative for hydroxyapatite (HA), alkaline phosphatase (ALP), and osteocalcin (OCN) (Fig. 1b(i)). In contrast, bmMSCs-derived tissues cultured in CHM or OCM were similarly rich in COL2A1 and ACAN (Fig. 1b(ii)), but when exposed to OCM, dark calcium salt deposits were detectable in brightfield images of the resulting tissues (Fig. 1a(ii)). These deposits corresponded to HA-positive areas in immunofluorescence staining (Fig. 1b(ii)). Moreover, a high expression of ALP and OCN was observed under both culture conditions (Fig. 1b(ii)). Immunofluorescence images at day 0 are presented in Fig. S1, confirming the undifferentiated state of both hACs and bmMSCs prior to on-chip culture.

We then analysed a set of osteochondrogenic and osteogenic marker genes to further assess the role of β-Gly in modulating hACs and bmMSCs differentiation (Fig. 1c). In hACs, exposure to OCM did not induce any change in the expression of chondrogenic markers (i.e., the *COL2A1/COL1A1* ratio and *ACAN*) or hypertrophic markers (*COL10A1* and *IHH*), compared to the standard CHM formulation. In contrast, for bmMSCs, switching from CHM to OCM resulted in a slight (non-statistically significant) decrease in the *COL2A1/COL1A1* ratio and a statistically significant reduction in *ACAN* expression when compared to both CHM-cultured bmMSCs and hACs. Clear differences between the two cell types were observed in the expression of hypertrophic genes, with statistically significantly lower *COL10A1* and *IHH* expression in hACs compared to bmMSCs cultured in either medium. The expression of bone-related markers bone sialoprotein 2 (*BSP2*), osteocalcin (*OCN*), *ALP*, and osteopontin (*OPN*) was consistently higher in bmMSCs compared to hACs and was not affected by OCM exposure in either cell type.

Overall, these results indicate that supplementation with β-Gly does not impair the chondrogenic potential nor induce hypertrophic differentiation of hACs, while it enhances the osteogenic and hypertrophic differentiation, as well as the mineralisation potential, of bmMSCs. The resulting OCM formulation was therefore selected to drive the simultaneous formation of mineralized and hyaline-like cartilaginous tissues in subsequent co-culture experiments.

### Introduction of a dual-compartment microscale device to directly visualize the interface between cartilaginous and mineralized 3D cellular constructs

Next, we introduced a new microscale device to enable the co-culture of adjacent hACs- and bmMSCs-based microtissues and the direct visualisation of their interface.

**Fig. 2:**
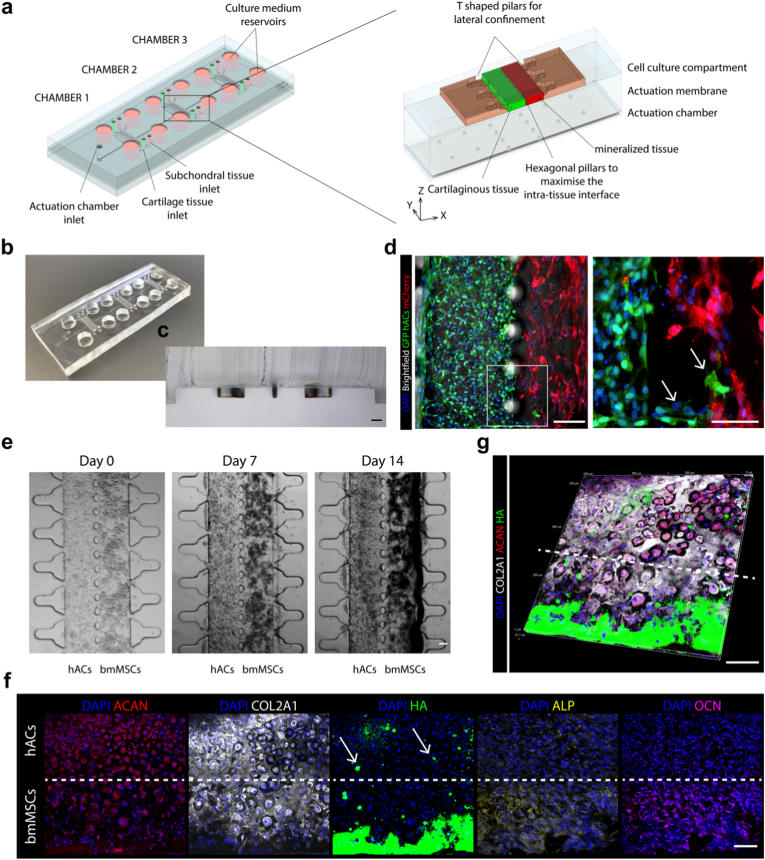
**Dual-compartment OoC device for hACs and bmMSCs co-culture. a.** 3D schematic of the dual-compartment device, comprising three independent culture chamber replicates. Each chamber includes two hydrogel channels for hACs- and bmMSCs-based constructs, depicted in green and red, respectively. **b.** Representative image of the fabricated device. Scale bar: 5 mm. **c.** Representative image of the cross-section (top layer only) of one of the three culture chambers. Scale bar: 100 μm. **d.** Immunofluorescence images of co-cultured constructs composed of GFP^+^ hACs and mCherry^+^ bmMSCs after 14 days of maturation in OCM. The right-hand image shows an inset focused on the tissue interface (n = 6 independently cultured constructs). The hexagonal pillar line separating the constructs is indicated by the dotted line. Migrating cells are marked by arrows. Scale bars: 100 μm. **e.** Representative brightfield images of hACs–bmMSCs co-cultures over time (N ≥ 6 biologically independent constructs from N = 3 donors per cell type). Scale bar: 100 μm. **f.** Immunofluorescence images of osteochondral constructs after 14 days of static maturation (n = 6 independently cultured constructs from ≥3 donors/experiments). Scale bar: 100 μm. The hexagonal pillar line separating the constructs is indicated by the dotted line. Arrows in the DAPI–HA staining highlight HA-positive cells in the hACs compartment. **g.** 3D confocal reconstruction of an osteochondral construct after 14 days of static differentiation. Scale bar: 100 μm. The hexagonal pillar line separating the constructs is indicated by the dotted line.

The dual-compartment device comprises three identical chambers, each featuring a four-channel configuration: two central hydrogel compartments for cell encapsulation, flanked by two lateral medium channels. Specifically, each 3D cell culture chamber includes two adjacent compartments for hosting cell-laden hydrogels (Fig. 2a, cartilaginous and mineralized tissues constructs are represented in green and red, respectively), separated by a row of hexagonal pillars designed to maximize the interface between the constructs. Each hydrogel compartment is further separated from its corresponding medium channel by a row of suspended T-shaped pillars. Fig. 2a provides an overview of the device layout and includes an inset of one of the cell culture chambers. Fig. 2b displays an example of a fabricated device, while Fig. 2c presents a cross-sectional image of one chamber, highlighting the four-channel configuration and the structural arrangement of the pillars separating the different channels.

To demonstrate the feasibility of generating bi-layered 3D constructs composed of directly interfaced cartilaginous and mineralized microtissues, GFP^+^ hACs- and mCherry^+^ bmMSCs-laden PEG-based hydrogels were injected into the flanked central compartments of the device and differentiated in OCM. After 14 days of co-culture, a clear separation between the two cell populations was maintained, with GFP^+^ hACs and mCherry^+^ bmMSCs remaining largely confined to their respective channels. A few migrating cells were visualised at the inter-tissue interface, suggesting the possible establishment of crosstalk (Fig. 2d; migrating cells are indicated by white arrows).

We then repeated the experiment using naïve hACs and bmMSCs (i.e., cells not expressing GFP or mCherry) to characterise the effect of the co-culture on the two populations. Clear differences between hACs-derived and bmMSCs-derived microtissues were evident in brightfield images, with dark mineralized areas appearing exclusively in the bmMSCs compartment as early as day 7 and becoming more pronounced by day 14 (Fig. 2e). Immunofluorescence imaging confirmed the formation of osteochondral-like constructs, comprising a cartilaginous layer (derived from hACs) positive for ACAN and COL2A1 (with COL2A1 positivity also retained in the central region of the bmMSCs-derived microtissue), and a mineralized layer (derived from bmMSCs) positive for HA, ALP, and OCN (Fig. 2f).

Notably, while hACs in monoculture remained completely negative for the HA staining (Fig. 1b(i)), HA-positive spots were detectable in the cartilaginous layer when co-cultured with bmMSCs (Fig. 2f, white arrows). Further evidence of mutual crosstalk between the two compartments was provided by the formation of a mineralisation gradient in the bmMSCs-based constructs. The portion of bmMSCs constructs immediately adjacent to the hACs compartment, while being positive for the early osteoblast markers ALP and OCN [35], remained rich in COL2A1 (Fig. 2f and g), thus being more similar to calcified cartilage. In contrast, areas farther from the hACs layer were strongly positive for HA and negative for COL2A1, indicating a more mature osteoblast phenotype (Fig. 2f and g). Notably, COL2A1 and HA positivity were almost mutually exclusive, as shown by evaluating the intensity of the two channels along lines extending from the hACs to the bmMSCs compartments (Fig. S2).

In summary, these data demonstrate the successful introduction of a dual-compartment microscale device that supports the co-culture of compartmentalized cartilaginous and mineralized microtissues over a 14-day period, enables direct observation of the inter-tissue interface, and promotes inter-tissue crosstalk.

### Characterization of hACs and bmMSCs phenotype in co-culture

**Fig. 3:**
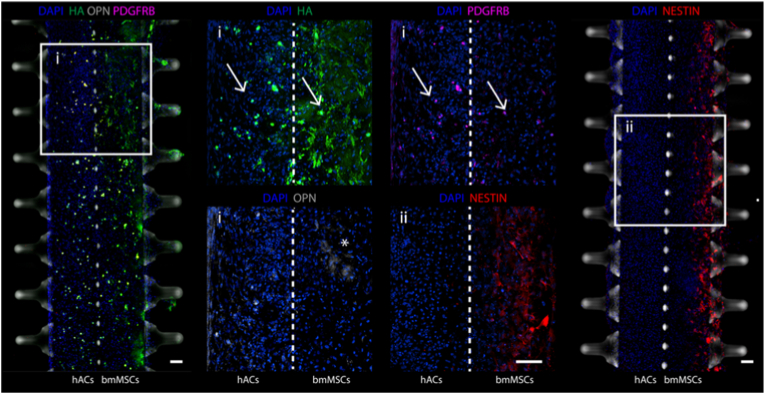
**Characterization of hACs and bmMSCs phenotypes in co-cultures.** Immunofluorescence images of hACs and bmMSCs co-cultured for 14 days in OCM (n ≥ 3 independently cultured samples from n = 3 hACs donors and n = 3 bmMSCs donors). Scale bar, 100 μm. PDGFRB^+^ cells colocalized with HA positive round cells in both hACs and bmMSCs compartments are indicated by white arrows. OPN^+^ areas (indicated by the white asterisk) were limited in extension but present and co-localized with HA positive regions. NESTIN^+^ cells were localized in the bmMSCs construct region further away from hACs. Areas marked with I and II represent insets from the lateral lower magnification images, where a darkened brightfield highlights the position of lateral and central pillars.

To further characterise the effect of the crosstalk between bmMSCs and hACs after 14 days of co-culture, we analysed the spatial distribution and expression of key phenotypic markers of osteochondral cells at the tissue interface (Fig. 3). Regions labelled (i) and (ii) in the low-magnification views correspond to high-resolution insets shown in the central panels, allowing detailed visualisation of marker localisation in relation to the microfluidic structure (with the interface indicated by the white dashed line).

Immunofluorescence staining revealed the presence of co-culture-dependent HA-positive round cells in both hACs and bmMSCs compartments, specifically in regions adjacent to the interface (indicated by white arrows). These cells co-localised with platelet-derived growth factor receptor beta (PDGFRB)-positive cells, a marker typically associated with mesenchymal skeletal stem cell progenitors [36]. Furthermore, in the bmMSCs compartment, we observed OPN-positive areas (indicated by white asterisks), which were spatially restricted and co-localised with HA-enriched regions, supporting the notion of early matrix remodelling and osteogenic signalling occurring at the interface. We also verified the presence of NESTIN positive cells (Fig. 3) in regions proximal to the external boarder of the bmMSCs constructs (i.e. far from the cartilage side), which we previously showed to be HA/ALP-positive (Fig. 2f and g). NESTIN is perivascular skeletal progenitor cells markers, which also identifies an ALP-positive osteoblast population in the epiphyses of mouse long bones [35]. Thus, co-culture appears to result in a stratified structure within the bmMSCs constructs, with a phenotype resembling calcified cartilage at the centre and a more mature osteoblastic phenotype towards the periphery.

Overall, this analysis demonstrates that the co-culture of hACs and bmMSCs within our dual-compartment OoC results in biphasic constructs exhibiting distinct spatial patterns of key osteochondral phenotypic markers. These findings indicate that the direct contact between hACs and bmMSCs modulates cell behaviour and differentiation phenotype at the microscale.

### Addition of a vascular compartment: bmMSCs assume a HUVECs-supporting phenotype upon co-culture and exposure to vascular inducing factors

Towards a modular increase in model complexity, we next aimed to incorporate a vascular cellular component (i.e., HUVECs) into the bmMSCs-based mineralized microtissue compartment.

Firstly, we optimized the cellular composition to promote the formation of vascularized and mineralized microtissues by establishing on-chip co-cultures of bmMSCs and RFP-expressing HUVECs at varying ratios (1:10, 1:2, and 3:2), while maintaining a total cell density of 50 × 10^6^ cells mL^−1^ within the PEG-based hydrogel. A lower density of 10 × 10^6^ cells mL^−1^ was also tested but proved too sparse to support sufficient cell–cell interaction or vascular structure formation in 3D, regardless of the cell ratio used (Fig. S3).

**Fig. 4:**
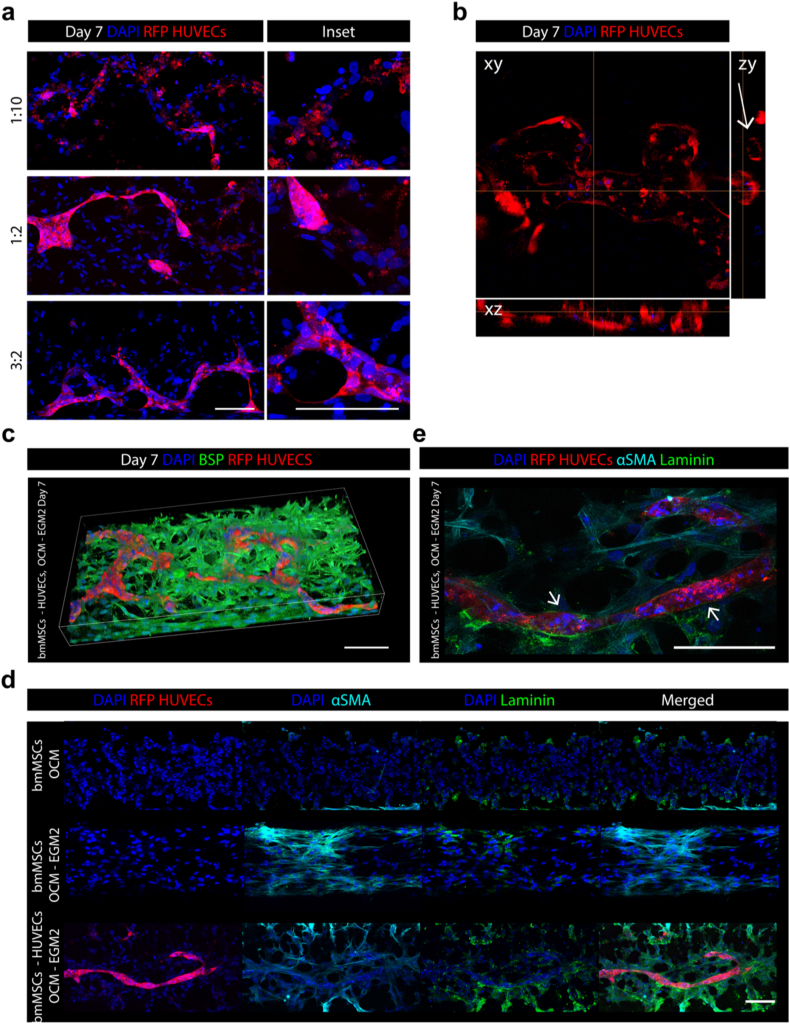
**Optimisation of the bmMSCs: HUVECs ratio in co-cultures to support the development of vascularized subchondral tissues.** On-chip co-cultures of bmMSCs and HUVECs were established at a total cell density of 50 × 10^6^ cells mL^−1^ within the hydrogel, using bmMSCs: HUVECs ratios of 1:10, 1:2, and 3:2. Co-cultures were maintained for 7 days in a 1:1 (v/v) mixture of OCM and EGM-2 (n = 4 independently cultured samples). **a.** Representative immunofluorescence images of co-cultures at day 7. RFP-expressing HUVECs enabled visualisation of the developing vascular networks. Insets on the right highlight how increasing the proportion of bmMSCs enhances the formation of vascular structures with a closed-vessel morphology. Scale bars, 100 μm. **b.** High-magnification immunofluorescence reconstruction of a vascular structure (bmMSCs: RFP-HUVECs ratio 3:2). The arrow in the ZY plane indicates a vessel with a lumen. Scale bar, 100 μm. **c.** Representative 3D confocal reconstruction of a bmMSCs: RFP-HUVECs (3:2) co-culture stained for bone sialoprotein (BSP, green) and counterstained with DAPI (blue). Scale bar, 100 μm. **d.** Immunofluorescence images comparing bmMSCs: RFP-HUVECs co-cultures and bmMSCs monocultures at day 7. Scale bar, 100 μm. **e.** 40 × magnification image of bmMSCs: RFP-HUVECs co-cultures at day 7. Arrows indicate laminin-rich structures encasing vascular elements. Scale bar, 100 μm.

After 7 days of co-culture in a 1:1 mixture of OCM and EGM-2, immunofluorescence analysis revealed that the 1:10 ratio failed to support the formation of well-defined vessel-like structures, resulting instead in sparse and loosely organised cellular assemblies. In contrast, higher proportions of bmMSCs (i.e., 1:2 and, in particular, 3:2) facilitated the development of clearly distinguishable vascular structures. The increased presence of bmMSCs promoted more defined, closed-loop vascular morphologies, as illustrated in the insets of Fig. 4a. Using 3D confocal reconstructions (Fig. 4b), we also visualised the emergence of vessel-like architectures characterised by luminal structures (Fig. 4b, ZY plane, bmMSCs: RFP-HUVECs 3:2).

Prolonging the culture time to 14 days resulted in an involution of the vessel-like structure, with the appearance of HUVECs shedding or detaching from the main vessels. At the same time, this prolonged culture revealed a reduced accumulation of COL2A1 and OCN compared to bmMSCs monocultures (Fig. S4). Nevertheless, osteogenic commitment was already evident by day 7, as indicated by the strong bone sialoprotein (BSP) staining throughout the tissue in the 3:2 ratio condition (Fig. 4c), which suggests early subchondral matrix deposition in the vascularized construct.

Further characterisation of the vascularized microconstructs at day 7 revealed the co-localisation of elongated cells expressing α-smooth muscle actin (α-SMA) with laminin-positive areas surrounding the vascular structures (Fig. 4d), indicative of a perivascular-like phenotype acquired by bmMSCs. Higher magnification imaging (Fig. 4e) confirmed the presence of laminin-rich sheaths encasing RFP^+^ vessels (indicated by arrows), mimicking features of native basement membranes. To determine whether this phenotype resulted specifically from interaction with endothelial cells or merely from exposure to the vascular-inducing medium EGM-2, we performed the same stainings on control conditions in which bmMSCs were monocultured within the same chip platform, either in OCM or in the 1:1 OCM: EGM-2 mixture. As shown in Fig. 4d, bmMSCs cultured for 7 days in OCM alone were negative for both α-SMA and laminin. In contrast, bmMSCs cultured in the OCM: EGM-2 mixture were positive for these markers, although the α-SMA and laminin structures appeared less organised.

Altogether, these findings demonstrate that increasing the bmMSCs content within the co-culture not only supports the formation of stable, lumenised microvessels, but also promotes early osteogenic commitment as early as day 7. In this context, the 3:2 ratio was identified as optimal for engineering vascularized and mineralized microtissues.

### Exploitation of the dual-compartment platform to model osteochondral crosstalk and inflammation-induced vascular invasion

Finally, all previously introduced components—namely, the cartilaginous construct and the vascularized, mineralized microtissue—were integrated within a single platform to investigate the interplay between engineered vascular and cartilage tissues.

**Fig. 5:**
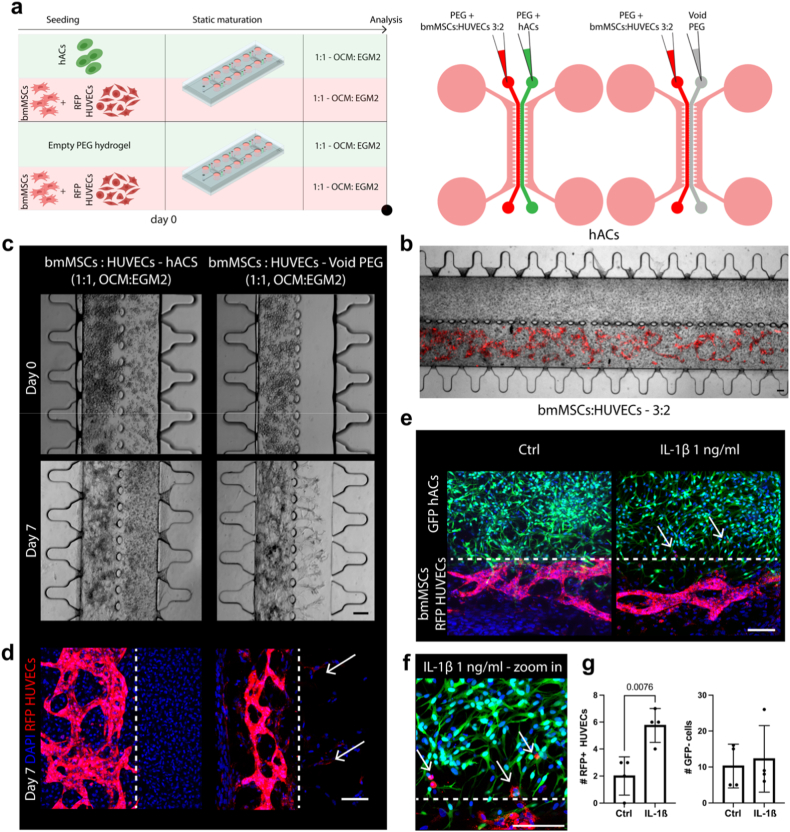
**Co-culture of hACs constructs with bmMSCs: RFP-HUVECs vascularized tissues. a.** Schematic of the experimental setup. Constructs were cultured in a 1:1 (v/v) mixture of OCM and EGM-2 for 7 days. bmMSCs: HUVECs co-cultures flanked by empty PEG hydrogels were used as controls, as highlighted by the diagrams on the right **b.** Representative image of a vascularized osteochondral construct at day 7. Brightfield and fluorescent images are overlaid. The use of RFP ^+^ HUVECs allowed to visualize the formation of a vascular network. Scale bar, 100 μm. **c.** Brightfield (day 0 and day 7) and **d.** immunofluorescence (day 7) images of complete osteochondral constructs and controls with empty PEG hydrogels instead of hACs. Arrows point to RFP^+^ HUVECs in the cartilaginous compartment (n = 4 independently cultured constructs per condition). Scale bars, 100 μm. **e.** Representative immunofluorescence images of osteochondral constructs made of GFP ^+^ hACs (cartilaginous construct) and bmMSCs: RFP^+^ HUVECs co-cultures (vascularized constructs) at day 7, with and without the administration of 1 ng ml^−1^ of IL-1β. Arrows point to RFP^+^ HUVECs in the cartilaginous compartment upon IL-1β administration (n = 4 independently cultured constructs per condition). The dotted line represents the pillar interface between the two compartments. Scale bar, 100 μm. **f.** Zoom-in of the picture in panel **e**. Arrows point to RFP ^+^ HUVECs in the cartilage compartment; the dotted line represents the pillar interface. Scale bar, 100 μm. **g.** Quantifications of HUVECs (RFP^+^), and of GFP^−^ cells (i.e., the sum of RFP^+^ HUVECs and RFP^−^ bmMSCs) in the cartilaginous compartment (n = 4 independently cultured constructs). Statistics by two tailed t-test for normal populations and by Mann–Whitney test for non-Gaussian populations. Populations normality was determined by Shapiro–Wilk test. P-values <0.05 are reported on the graph.

To this end, hACs were co-cultured alongside vascularized bmMSCs: HUVECs microtissues within the dual-compartment chip (Fig. 5a). Cultures were maintained for 7 days in a 1:1 mixture of OCM and EGM-2. The use of RFP-labelled HUVECs enabled direct visualisation of vascular network formation in the subchondral compartment (Fig. 5b, lower channel), with well-defined vascular architectures evident within the hydrogel core by day 7. As shown in Fig. 5c, the presence of hACs-based constructs markedly influenced vascular distribution and the invasive behaviour of bmMSCs: HUVECs co-cultures compared to void controls (i.e., cultures where bmMSCs: HUVECs co-cultures were flanked by empty PEG hydrogels). By day 7, immunofluorescence imaging confirmed the invasive phenotype of RFP labelled HUVECs and bmMSCs in the absence of hACs, with both RFP^+^ (HUVECs) and RFP^−^ (bmMSCs) cells migrating into the initially empty PEG gel (arrows). In contrast, constructs containing hACs maintained a more compartmentalized and spatially confined organisation (Fig. 5d).

To investigate the system’s responsiveness to an inflammatory stimulus (inflammation being a key mediator of OA pathogenesis [37]), constructs made with GFP^+^ hACs, RFP^+^ HUVECs, and naïve bmMSCs were exposed to 1 ng ml^−1^ of IL-1β. As illustrated in Fig. 5e and f, while the overall appearance of the HUVECs vascular network remained unchanged (Fig. S5), IL-1β caused a decrease in hACs capacity to resist the migration tendency of RFP^+^ HUVECs and RFP^−^ bmMSCs into the cartilage region (migrating RFP^+^ HUVECs are indicated by arrows). Quantification of RFP^+^ cells within the cartilage compartment (Fig. 5g) confirmed increased HUVEC infiltration following IL-1β treatment, validating the model’s capacity to capture inflammation-driven alterations in osteochondral crosstalk.

Collectively, these findings highlight the suitability of our dual-compartment platform for studying vascular and stromal invasion dynamics under both homeostatic and inflammatory conditions. Notably, its modular design allows precise attribution of cell-type-specific contributions, offering a powerful tool to dissect complex cellular crosstalk in engineered osteochondral systems.

## Discussion

In this study, we introduced a dual-compartment OoC platform designed to “deconstruct and reconstruct” the vascularized bone–cartilage interface in a modular *in vitro* setup.

Early OoC models for OA research have been primarily aimed at replicating the cartilaginous tissue[[12], [13], [14], [15]]. However, OA is increasingly recognized as a whole-joint disease [4] and understanding the cause-effect mechanisms underlying its aetiology and progression requires analysing the complex interplay among different joint tissues. For instance, whether OA originates in cartilage or in the underlying subchondral bone remains a topic of active debate. Furthermore, OA progression involves the reactivation of vascularization programs characterizing the cartilage-to-bone transition in embryonic development, bone fracture repair, and adolescent growth [38]. More comprehensive, yet modular, vascularized osteochondral models are therefore needed to elucidate the mechanisms driving OA pathogenesis.

To meet this need, microscale osteochondral models (with [8] or without [39] vascular components) and multi-tissue systems [16] have been developed, relying on millimetre-scale bioreactors engineered to selectively direct mesenchymal stromal cells (e.g., bmMSCs) toward both cartilage and subchondral bone tissue phenotypes. Nevertheless, bmMSCs are transcriptionally different from articular chondrocytes [40,41] and intrinsically primed towards endochondral ossification [23]. Therefore, while bmMSCs’ intrinsic commitment can be used to model osteogenic mineralized tissues, hACs might represent a more suitable cell source for replicating the transcriptomic variability of the cartilaginous compartment [42].

To validate this hypothesis, we took advantage of a previously published single compartment OoC platform (uBeat® Compress Platform, BiomimX® S.r.l.) [15], and independently assessed the chondrogenic and osteogenic potential of hACs- and bmMSCs-derived microconstructs. For this purpose, the microconstructs were either cultured in an established chondrogenic medium [34] – i.e. CHM – or in OCM, i.e. CHM further supplemented with β-Gly, a source of inorganic phosphate previously reported to be involved in HA deposition and bone formation [43]. When bmMSCs were cultured in OCM, we observed enhanced mineral deposition as compared to constructs exposed to the standard CHM. In contrast, the same medium supplied to hACs-based microconstructs resulted in a stable chondrogenic phenotype, without induction of hypertrophy or mineralisation. The proposed OCM led to an upregulation over time of genes connected with chondrogenesis (i.e., *COL2A1* and *ACAN*) at a similar level in both hACs- and bmMSCs-derived tissues. Conversely, the expression of genes associated with hypertrophic differentiation (i.e., *COL10A1*, *IHH*) and bone formation (i.e., *ALP*, *OCN*, *OPN*, *BSP2*) was up to two orders of magnitude higher in bmMSCs-based constructs compared with hACs, independently from the culture medium formulation.

Moreover, in accordance with the intrinsic capacity of bmMSCs to acquire (pre)hypertrophic traits in response to pro-chondrogenic factors [23], accumulation of OCN and ALP was detected bmMSCs cultured in both OCM (higher) and CHM (milder) formulations.

Thus, by exploiting the respective intrinsic commitment of hACs and bmMSCs toward articular chondrogenesis and endochondral ossification, a single medium formulation (i.e., the OCM) could be used to form flanked bone-cartilage microtissues with a direct interface by introducing a purposefully engineered dual-compartment microfluidic chip. This approach avoided the need for more complex compartmentalized culturing systems, which require continuous flow to provide different medium compositions to distinct cell sources or to achieve morphogen gradients to differentiate a single cell source into different cell types [10].

From a technological perspective, the side-by-side disposition of our dual-compartment device enabled direct observation of both tissues and their interface. Additionally, the platform design allowed us to (i) replicate the shape of the external pillars and the construct dimensions of the uBeat® Compress Platform, facilitating direct comparisons between devices, and (ii) maximize the contact area between the two cell compartments due to the presence of a pure interface beneath the pillars, thereby promoting direct cell interaction.

Such double-compartment device enabled to obtain bi-layer microtissues that maintain a well-defined but interactive interface between the forming cartilaginous and mineralized constructs. In fact, while a clear boundary between the two tissue was maintained for 14 days, as visualised using GFP^+^ hACs and mCherry^+^ bmMSCs, the detection of HA positive cells in the cartilage compartment and a non-symmetrical mineralisation pattern in the bone compartment indicated bidirectional influence between the two tissues (features which would be difficult to resolve in stacked or millimetre-scale *in vitro* models). The presence of a bi-directional crosstalk was also highlighted by the spatial mapping of NESTIN, PDGFRB, and OPN, which showed how the bmMSCs’ phenotype is microenvironment-dependent and influenced by the presence of hACs. Previous findings linked NESTIN expression to ALP^+^ osteoblasts in the epiphyses of mouse long bones [35]. The localisation of NESTIN^+^ bmMSCs at the external mineralized border of the constructs, distant from the cartilage layer, suggests that NESTIN expression marks a differentiated osteoblast-like phenotype, in contrast to the more calcified cartilage phenotype observed in bmMSCs directly contacting the cartilage compartment. PDGFRB is involved in bmMSCs fate specification and has been associated with skeletal stem cell progenitors [36]. The detection of PDGFRB^+^ cells in the hACs compartment, which are also positive for OPN and HA, might therefore indicate the presence of progenitor-like chondrocytes that differentiate upon co-culture with the bmMSCs compartment. This hypothesis, supported by previous reports of cartilage progenitors with roles in OA development [44], is strengthened by the fact that very limited bmMSCs migration was detected deep within the cartilage compartment, excluding the possibility that PDGFRB^+^ cells are migrating bmMSCs.

Given the relevance of the vascular component in osteochondral physiology and OA progression, we then integrated endothelial cells into the mineralized compartment. By titrating the bmMSCs:HUVECs ratio, we identified a 3:2 co-culture at high cell density (50 × 10^6^ cells ml^−1^) as optimal for forming lumenized, α-SMA/laminin-stabilized microvessels within one week. This condition supported both bmMSCs’ early osteogenic commitment, as indicated by BSP2 expression, and their rapid acquisition of a perivascular phenotype, as shown by the presence of α-SMA-expressing cells surrounding the vessels. A lower cellular density (10 × 10^6^ cells ml^−1^), similar to the one used in other OoC models [21,45], was also tested; however, under our conditions, it yielded sparse, disorganized structures with no clear microvessel formation. This discrepancy may stem from the use of a non-RGD-functionalized PEG-based hydrogel in our setup, optimized to allow chondrogenesis by competent cells [46], as opposed to the fibrin matrices [47] or RGD-functionalized PEG hydrogels [48] typically used to support vascularization. Conversely, the observation that a 7-day culture period was optimal for the formation and maintenance of compact vascular structures—whereas vessel regression was observed after 14 days in culture—aligns with previous reports demonstrating that fluid flow and the associated shear stresses are essential for the long-term maintenance of vascular networks in OoC systems [49,50]. Future developments of our platform could therefore incorporate lumenized lateral channels with constant fluid flow to sustain the long-term culture of the vascular network in the bone compartment.

Notably, the adoption of single-culture controls was instrumental in ruling out medium composition as the sole driver of bmMSCs adopting a perivascular supporting phenotype, implicating direct bmMSCs–endothelial interactions and matrix cues as critical for early vessel maturation. Single bmMSCs cultures supplemented with the OCM:EGM-2 mixture showed no perivascular-like patterning in α-SMA and laminin structures, supporting the conclusion that bmMSCs’ acquisition of a perivascular supporting phenotype depends on direct bmMSCs–endothelial interaction and is not merely a response to angiogenic soluble factors. These findings underscore the critical role of cell–cell contact and microenvironmental architecture in orchestrating functional vascular organization in engineered constructs.

Lastly, we showed that cartilage acts as an effective anti-invasion barrier under homeostatic conditions, and that this barrier can be disrupted by IL-1β. Without inflammation, the hACs compartment sharply limited endothelial and stromal invasion, whereas empty hydrogel controls permitted unrestricted migration of both bmMSCs and HUVECs. These findings confirm that the model mirrors the capacity of cartilage to remain avascular by exerting an inhibitory effect on vascular and stromal invasion at a healthy osteochondral interface [51]. IL-1β exposure reversed such behaviour, permitting endothelial entry into the cartilaginous layer, in line with clinical evidence that inflammatory upregulation coincides with cartilage vascular penetration in OA [51,52]. Although in our study IL-1β was administered at a supraphysiological concentration (1 ng ml^−1^) if compared to the levels typically found in the synovial fluid of OA patients [53], this dose is recognized as a standard *in vitro* inflammatory stimulus and it is consistently used to reproducibly induce OA-like catabolic responses [15,21].

Finally, a few important considerations regarding the scope and limitations of our approach must be addressed. We deliberately adopted a reductionist, bottom-up strategy, in which the behaviour and contribution of each cellular component are evaluated separately before increasing the model’s complexity. In contrast, other recent osteochondral-on-chip devices have incorporated a broader range of cell types—such as osteoblasts, osteoclasts, endothelial cells, and mesenchymal stem cells in the subchondral layer, alongside chondrocytes in the cartilaginous layer [21]. While such complex systems may be better suited for studying holistic responses of the osteochondral unit to therapeutic interventions, their complexity often makes it difficult to attribute observed phenomena to specific cell types or secreted factors. Moreover, these models frequently rely on multilayer PDMS structures and user-dependent assembly procedures, which can hinder reproducibility and limit the resolution of cell-specific analyses.

Our single-layer device operates under static conditions and requires only three well-defined human cell types and yet it maintains the ability to recapitulate key OA hallmarks such as vascular invasion and early matrix calcification. As such, our platform strikes a pragmatic balance between biological relevance and experimental tractability, making it particularly suitable for mechanistic studies.

Despite these advantages, future work should explore how vascular invasion is influenced by mechanical loading—an established risk factor for OA [2]. Moreover, a more comprehensive joint-on-chip model should include additional cell types such as synoviocytes and immune cells, both of which are critically involved in OA onset and progression [54]. Importantly, in this context, our dual-compartment device is already engineered to support 30 % hyperphysiological mechanical stimulation, a level previously shown to induce OA-like phenotypes in both cartilaginous [15] and osteochondral [46] on-chip models, and it is fully compatible with previously reported strategies for integrating immune [45] and synovial [55] cells into organ-on-chip systems.

## Conclusion

In summary, we showcased the potential of an OoC-based, bottom-up approach for dissecting the contribution of individual factors within a complex biological system, such as the multicellular crosstalk at the cartilage–bone interface. By treating the key cellular components of cartilage, mineralized tissue, and endothelium as plug-and-play modules, we demonstrated that introducing or removing cues on demand (e.g., the vasculature) enables detailed investigation of how individual cell types commit to specific lineages in monoculture or co-culture conditions, as well as how their functional behaviour changes in response to defined stimuli.

Following this approach, we: (i) established common culture conditions for generating cartilaginous and mineralized microscale tissues starting, respectively, from hACs and bmMSCs; (ii) integrated these into bi-layered constructs using a newly designed dual-compartment OoC; (iii) identified a bmMSCs: HUVECs ratio that promotes the formation of lumenized, α-SMA/laminin-stabilized microvessels within the mineralized tissue; and (iv) leveraged the observation window provided by the dual-compartment OoC to visualize how hACs inhibit vascular invasion at the cartilage–subchondral tissue interface. Notably, this barrier effect was compromised by IL-1β, which reduced the capacity of hACs to prevent pathological cell migration and invasion as observed in clinical settings.

By enabling optical access and modular, stepwise assembly of cartilaginous, mineralized, and vascularized components, this dual-compartment Organ-on-Chip provides a powerful tool for dissecting the sequence of events that govern vascular invasion and matrix remodelling in OA. Its simplicity and scalability make it well-suited for future high-content mechanistic studies and for targeted screening of therapies aimed at preserving the cartilage–bone interface.

## Funding

This work was supported by Cariplo Foundation (grant number #2021-1564 to POC) and by the 10.13039/501100001711Swiss National Science Foundation (grant number 310030_175660/1, to ABA).

## Author contribution

AMA and MRA conceived the dual compartment OoC design. AMA produced the device, planned and executed biological experiments, and performed all analyses. MEH fabricated and provided the hydrogel. AMA, ABA, MRA, POC and IMA conceived the project. POC and AMA wrote the manuscript with the contribution of ABA and IMA. AMA realized the manuscript figures. All authors discussed the results, commented on the manuscript, and contributed to its final version. POC and IMA contributed equally to this work.

## Conflict of interest

Paola Occhetta and Marco Rasponi share equities in BiomimX S.r.l.

## Data Availability

The authors declare that all data supporting the findings of this study are available within the paper and its Supplementary Information file; further specifications are available from the corresponding authors upon reasonable request.

## References

[1] Goldring S.R., Goldring M.B. (2016). Changes in the osteochondral unit during osteoarthritis: structure, function and cartilage–bone crosstalk. Nat Rev Rheumatol.

[2] Sanchez-Adams J., Leddy H.A., McNulty A.L., O’Conor C.J., Guilak F. (2014). The mechanobiology of articular cartilage: bearing the burden of osteoarthritis. Curr Rheumatol Rep.

[3] Findlay D.M., Kuliwaba J.S. (2016). Bone-cartilage crosstalk: a conversation for understanding osteoarthritis. Bone Res.

[4] Loeser R.F., Goldring S.R., Scanzello C.R., Goldring M.B. (2012). Osteoarthritis: a disease of the joint as an organ. Arthritis Rheum.

[5] Chen Y., Hu Y., Yu Y.E., Zhang X., Watts T., Zhou B. (2018). Subchondral trabecular rod loss and plate thickening in the development of osteoarthritis. J Bone Miner Res.

[6] Kilian D., Ahlfeld T., Akkineni A.R., Bernhardt A., Gelinsky M., Lode A. (2020). 3D bioprinting of osteochondral tissue substitutes – in vitro-chondrogenesis in multi-layered mineralized constructs. Sci Rep.

[7] Idaszek J., Costantini M., Karlsen T.A., Jaroszewicz J., Colosi C., Testa S. (2019). 3D bioprinting of hydrogel constructs with cell and material gradients for the regeneration of full-thickness chondral defect using a microfluidic printing head. Biofabrication.

[8] Pirosa A., Gottardi R., Alexander P.G., Puppi D., Chiellini F., Tuan R.S. (2021). An in vitro chondro-osteo-vascular triphasic model of the osteochondral complex. Biomaterials.

[9] Armstrong J.P.K., Pchelintseva E., Treumuth S., Campanella C., Meinert C., Klein T.J. (2022). Tissue engineering cartilage with deep zone cytoarchitecture by high-resolution acoustic cell patterning. Adv Healthcare Mater.

[10] Smith K.W.Y., Fung S.L., Wu H.F., Chiesa I., Vozzi G., De Maria C. (2025). Developing an in vitro osteochondral micro-physiological system for modeling cartilage-bone crosstalk in arthritis. Front Immunol.

[11] Ingber D.E. (2022). Human organs-on-chips for disease modelling, drug development and personalized medicine. Nat Rev Genet.

[12] Rosser J., Bachmann B., Jordan C., Ribitsch I., Haltmayer E., Gueltekin S. (2019). Microfluidic nutrient gradient–based three-dimensional chondrocyte culture-on-a-chip as an in vitro equine arthritis model. Mater Today Bio.

[13] Lee D., Erickson A., You T., Dudley A.T., Ryu S. (2018). Pneumatic microfluidic cell compression device for high-throughput study of chondrocyte mechanobiology. Lab Chip.

[14] Paggi C.A., Venzac B., Karperien M., Leijten J.C.H., Le Gac S. (2020). Monolithic microfluidic platform for exerting gradients of compression on cell-laden hydrogels, and application to a model of the articular cartilage. Sensor Actuator B Chem.

[15] Occhetta P., Mainardi A., Votta E., Vallmajo-Martin Q., Ehrbar M., Martin I. (2019). Hyperphysiological compression of articular cartilage induces an osteoarthritic phenotype in a cartilage-on-a-chip model. Nat Biomed Eng.

[16] Li Z., Lin Z., Liu S., Yagi H., Zhang X., Yocum L. (2022). Human mesenchymal stem cell-derived miniature joint system for disease modeling and drug testing. Adv Sci.

[17] Rothbauer M., Byrne R.A., Schobesberger S., Calvo I.O., Fischer A., Reihs E.I. (2021). Establishment of a human three-dimensional chip-based chondro-synovial coculture joint model for reciprocal cross talk studies in arthritis research. Lab Chip.

[18] Mondadori C., Palombella S., Salehi S., Talo G., Visone R., Rasponi M. (2021). Recapitulating monocyte extravasation to the synovium in an organotypic microfluidic model of the articular joint. Biofabrication.

[19] H L., Tp L., Pg A., R G., Rs T. (2014). Stem cell-based microphysiological osteochondral system to model tissue response to interleukin-1β. Mol Pharm.

[20] Tuerlings M., Boone I., Eslami Amirabadi H., Vis M., Suchiman E., van der Linden E. (2022). Capturing essential physiological aspects of interacting cartilage and bone tissue with osteoarthritis pathophysiology: a human osteochondral Unit-on-a-Chip model. Adv Mater Technol.

[21] Salehi S., Brambilla S., Rasponi M., Lopa S., Moretti M. (2024). Development of a microfluidic vascularized osteochondral model as a drug testing platform for osteoarthritis. Adv Healthcare Mater.

[22] Shi X., Zhou J., Zhao Y., Li L., Wu H. (2013). Gradient-regulated hydrogel for interface tissue engineering: steering simultaneous osteo/chondrogenesis of stem cells on a chip. Adv Healthcare Mater.

[23] Occhetta P., Pigeot S., Rasponi M., Dasen B., Mehrkens A., Ullrich T. (2018). Developmentally inspired programming of adult human mesenchymal stromal cells toward stable chondrogenesis. Proc Natl Acad Sci U S A.

[24] Ong L.J.Y., Sun A.R., Wang Z., Lee J., Prasadam I., Toh Y.C. (2024). Localized oxygen control in a microfluidic osteochondral interface model recapitulates bone–cartilage crosstalk during osteoarthritis. Adv Funct Mater.

[25] Conceição F., Meneses J., Lebre F., Becker M., Araújo-Gomes N., Vos R. (2025). Sex-stratified osteochondral organ-on-chip model reveals sex-specific responses to inflammatory stimulation. Mater Today Bio.

[26] Hatzikotoulas K., Southam L., Stefansdottir L., Boer C.G., McDonald M.-L., Pett J.P. (2025 2025). Translational genomics of osteoarthritis in 1,962,069 individuals. Nature.

[27] Scotti C., Piccinini E., Takizawa H., Todorov A., Bourgine P., Papadimitropoulos A. (2013). Engineering of a functional bone organ through endochondral ossification. Proc Natl Acad Sci.

[28] Hu Y., Chen X., Wang S., Jing Y., Su J. (2021). Subchondral bone microenvironment in osteoarthritis and pain. Bone Res.

[29] Roehm K.D., Chiesa I., Haithcock D., Gottardi R., Prabhakarpandian B. (2025). A vascularized microfluidic model of the osteochondral unit for modeling inflammatory response and therapeutic screening. Lab Chip.

[30] Evans Nicholas D., Minelli Caterina, Gentleman Eileen, LaPointe Vanessa, Patankar Sameer N., Kallivretaki Maria (2009). Substrate stiffness affects early differentiation events in embryonic stem cells. Eur Cell Mater.

[31] Ehrbar M., Rizzi S.C., Schoenmakers R.G., San Miguel B., Hubbell J.A., Weber F.E. (2007). Biomolecular hydrogels formed and degraded via site-specific enzymatic reactions. Biomacromolecules.

[32] Pu Y., Gingrich J., Veiga-Lopez A. (2021). A 3-Dimensional microfluidic platform for modeling human extravillous trophoblast invasion and toxicological screening. Lab Chip.

[33] Miot S., Gianni-Barrera R., Pelttari K., Acharya C., Mainil-Varlet P., Juelke H. (2009). In vitro and in vivo validation of human and goat chondrocyte labeling by green fluorescent protein lentivirus transduction. Tissue Eng C Methods.

[34] Acevedo Rua L., Mumme M., Manferdini C., Darwiche S., Khalil A., Hilpert M. (2021). Engineered nasal cartilage for the repair of osteoarthritic knee cartilage defects. Sci Transl Med.

[35] Coutu D.L., Kokkaliaris K.D., Kunz L., Schroeder T. (2017). Three-dimensional map of nonhematopoietic bone and bone-marrow cells and molecules. Nat Biotechnol.

[36] Sivaraj K.K., Jeong H.W., Dharmalingam B., Zeuschner D., Adams S., Potente M. (2021). Regional specialization and fate specification of bone stromal cells in skeletal development. Cell Rep.

[37] Robinson W.H., Lepus C.M., Wang Q., Raghu H., Mao R., Lindstrom T.M. (2016). Low-grade inflammation as a key mediator of the pathogenesis of osteoarthritis. Nat Rev Rheumatol.

[38] Whelan I.T., Burdis R., Shahreza S., Moeendarbary E., Hoey D.A., Kelly D.J. (2023). A microphysiological model of bone development and regeneration. Biofabrication.

[39] Lin H., Lozito T.P., Alexander P.G., Gottardi R., Tuan R.S. (2014). Stem cell-based microphysiological osteochondral system to model tissue response to interleukin-1?. Mol Pharm.

[40] Frerker N., Karlsen T.A., Stensland M., Nyman T.A., Rayner S., Brinchmann J.E. (2023). Comparison between articular chondrocytes and mesenchymal stromal cells for the production of articular cartilage implants. Front Bioeng Biotechnol.

[41] Sebastian A., McCool J.L., Hum N.R., Murugesh D.K., Wilson S.P., Christiansen B.A. (2021). Single-cell RNA-seq reveals transcriptomic heterogeneity and post-traumatic osteoarthritis-associated early molecular changes in mouse articular chondrocytes. Cells.

[42] Mainardi A., Börsch A., Occhetta P., Ivanek R., Ehrbar M., Krattiger L. (2025). An organ-on-chip platform for strain-controlled, tissue-specific compression of cartilage and mineralized osteochondral interface to study mechanical overloading in osteoarthritis. Adv Healthcare Mater.

[43] Chang Y.-L., Stanford C.M., Keller J.C. (2000). Calcium and phosphate supplementation promotes bone cell mineralization: implications for hydroxyapatite (HA)-enhanced bone formation. J Biomed Mater Res.

[44] Jiang | April, Tuan Y.S. (2014). Origin and function of cartilage stem/progenitor cells in osteoarthritis. Nat Rev Rheumatol.

[45] Mondadori C., Palombella S., Salehi S., Talò G., Visone R., Rasponi M. (2021). Recapitulating monocyte extravasation to the synovium in an organotypic microfluidic model of the articular joint. Biofabrication.

[46] Mainardi A., Börsch A., Occhetta Paola, Occhetta P., Ivanek R., Ehrbar M. (2025). An organ-on-chip platform for strain-controlled, tissue-specific compression of cartilage and mineralized osteochondral interface to study mechanical overloading in osteoarthritis. Adv Healthcare Mater.

[47] Park Y.K., Tu T.Y., Lim S.H., Clement I.J.M., Yang S.Y., Kamm R.D. (2014). In vitro microvessel growth and remodeling within a three-dimensional microfluidic environment. Cell Mol Bioeng.

[48] Blache U., Vallmajo‐Martin Q., Horton E.R., Guerrero J., Djonov V., Scherberich A. (2018). Notch‐inducing hydrogels reveal a perivascular switch of mesenchymal stem cell fate. EMBO Rep.

[49] Quintard C., Tubbs E., Jonsson G., Jiao J., Wang J., Werschler N. (2024). A microfluidic platform integrating functional vascularized organoids-on-chip. Nat Commun.

[50] Pollet A.M.A.O., den Toonder J.M.J. (2020). Recapitulating the vasculature using organ-on-chip technology. Bioengineering.

[51] Deng B., Chen C., Gong X., Guo L., Chen H., Yin L. (2017). Chondromodulin-I expression and correlation with angiogenesis in human osteoarthritic cartilage. Mol Med Rep.

[52] Oegema T.R., Carpenter R.J., Hofmeister F., Thompson R.C. (1997). The interaction of the zone of calcified cartilage and subchondral bone in osteoarthritis. Microsc Res Tech.

[53] Tsuchida A.I., Beekhuizen M., t Hart M.C., Radstake T.R.D.J., Dhert W.J.A., Saris D.B.F. (2014). Cytokine profiles in the joint depend on pathology, but are different between synovial fluid, cartilage tissue and cultured chondrocytes. Arthritis Res Ther.

[54] Chou C.-H., Jain V., Gibson J., Attarian D.E., Haraden C.A., Yohn C.B. (2020). Synovial cell cross-talk with cartilage plays a major role in the pathogenesis of osteoarthritis. Sci Rep.

[55] Palma C., Aterini B., Catozzi C., Nezi L., Lopa S., Manzo T. (2024). A compartmentalized microfluidic platform to investigate immune cells cross-talk in rheumatoid arthritis. Biofabrication.

