# Supplementary material for: Modular de- and re-construction of vascularized osteochondral tissues in an Organ-on-Chip dual-compartment platform
Source: J Orthop Translat. 2025 Nov 27;56:101017. doi: 10.1016/j.jot.2025.10.009 (PMC12988498; doi:10.1016/j.jot.2025.10.009)
Supplement: Multimedia component 1 [file mmc1.docx]

**Modular de- and re-construction of vascularized osteochondral tissues in an Organ-on-Chip dual-compartment platform**

Andrea Mainardi^a^, Andrea Barbero^a^, Martin Ehrbar^b^, Marco Rasponi^c^, Ivan Martin^a#, *^, Paola Occhetta^c, d#^

***Corresponding author**

^a^ Department of Biomedicine, University Hospital Basel, University of Basel, Hebelstrasse 20, 4031 Basel, Switzerland.

^b^ Department of Obstetrics, University Hospital Zurich, Frauenklinikstrasse 10, 8091, Zurich, Switzerland, and Zurich Centre for Integrative Human Physiology, Winterthurerstrasse 190, 8057 Zürich, Switzerland.

^c^ Department of Electronics, Information and Bioengineering, Politecnico di Milano, Via Golgi 39, 20133 Milan, Italy.

^d^ BiomimX S.r.l., Viale Decumano 41, 20157, Milan, Italy.

E-Mail: [ivan.martin@usb.ch](mailto:ivan.martin@usb.ch)

^#^equally contributing authors

Table of Contents

[Figure S1: Day 0 controls for hACs and bmMSCs differentiation stainings. 2](#_Toc209980334)

[Figure S2. Collagen type II and hydroxyapatite distribution in hACs-bmMSCs co-cultures. 2](#_Toc209980335)

[Figure S3. Establishment of bmMSCs and HUVECs co-cultures on-chip. 3](#_Toc209980336)

[Figure S4. bmMSCs and HUVECs co-cultures at day 14. 4](#_Toc209980337)

[Figure S5. Overview of vascular structures upon IL-1β administration. 4](#_Toc209980338)

[Table S1: Demographics of hACs and bmMSCs donors used in this study. 5](#_Toc209980339)


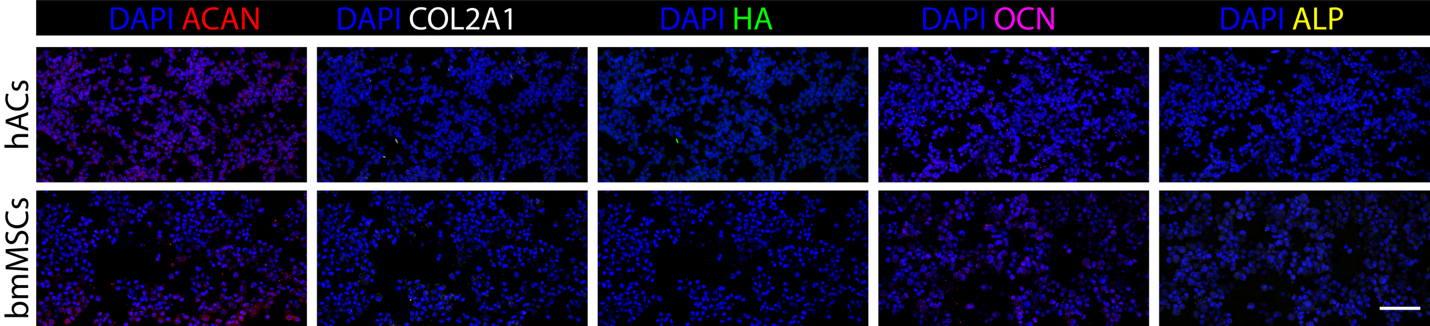


**Figure S1: Day 0 controls for hACs and bmMSCs differentiation stainings.** Immunofluorescence images of hACs and bmMSCs constructs at day 0 (n≥3 biologically independent samples from n≥3 donors for each condition). Scale bar, 100 µm.


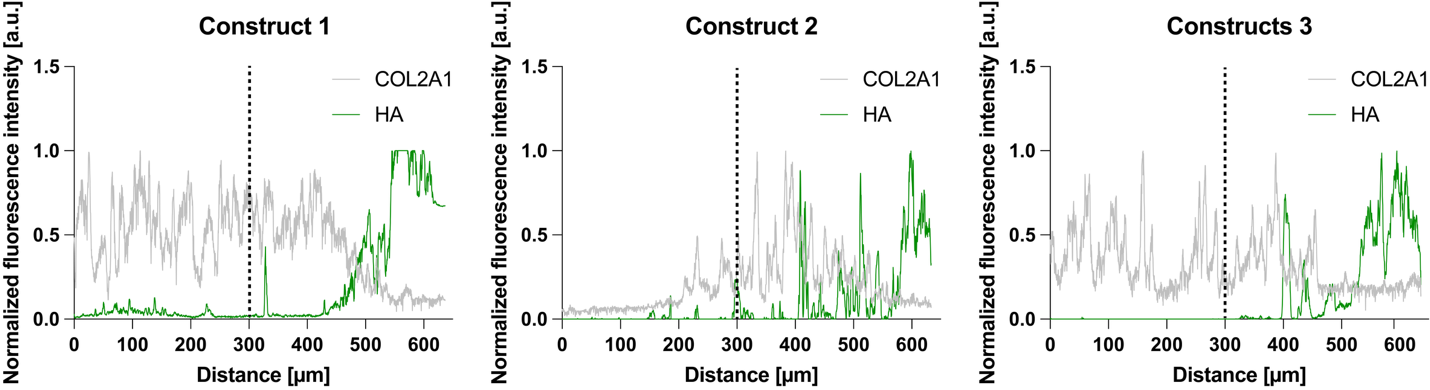


**Figure S2. Collagen type II and hydroxyapatite distribution in hACs-bmMSCs co-cultures.** Representative line plots of the normalized intensity profile across the osteochondral interface of hACs-bmMSCs constructs (as depicted in figure 2f). "0" indicates the outer border of the hACs constructs, "600" marks the outer layer of the bmMSCs layer. The dotted line denotes the nominal position of the hexagonal pillars separating the two cell-laden hydrogels. The normalized intensity was calculated by dividing all fluorescence intensity values by the highest measured intensity value in each fluorescence channel (n = 3 independently cultured constructs from *n* = 3 donors/experiments). For each construct, line plots were calculated at three separate locations and results were averaged.


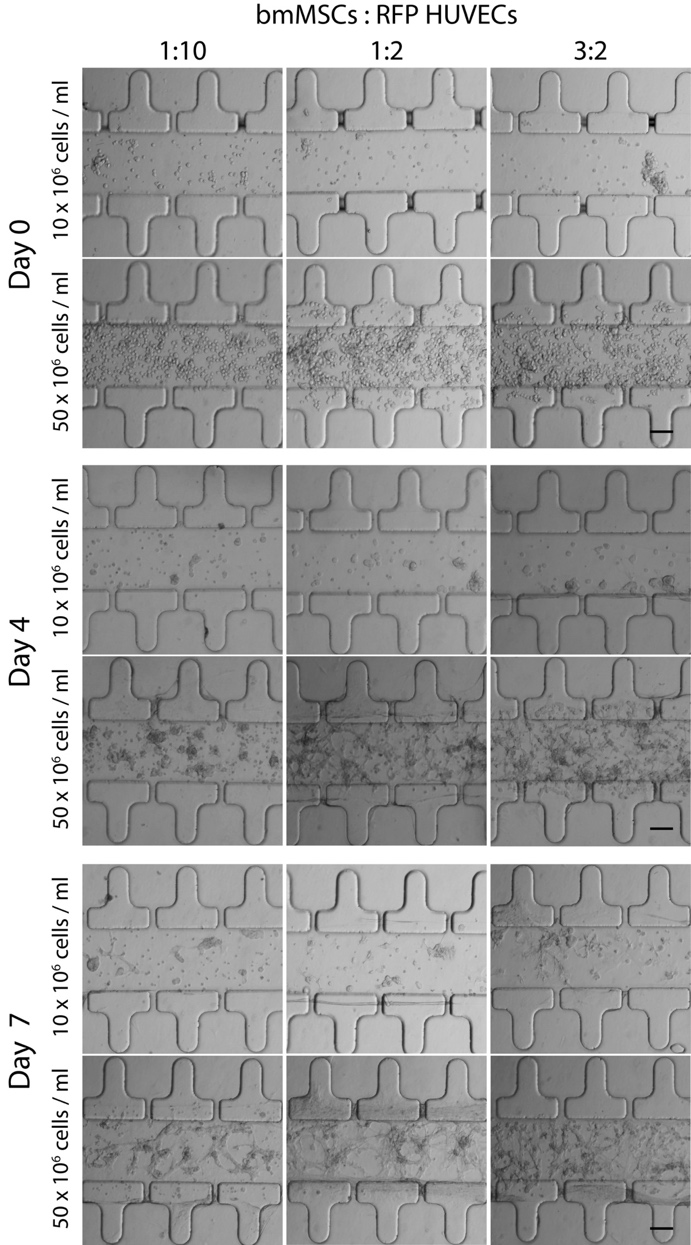


**Figure S3. Establishment of bmMSCs and HUVECs co-cultures on-chip**. Representative brightfield pictures of bmMSCs: HUVECs co-cultures over time. Total cellular densities in the hydrogel of 10 x 10^6^ cells ml^-1^ and 50 x 10^6^ cells ml^-1^ were considered; bmMSCs: HUVECs ratios of, respectively, 1:10, 1:2, and 3:2 were analysed. Co-cultures were performed statically, for 7 days, and using a 1:1 (v/v) mixture of OCM and EGM-2 (n=3 biologically independent constructs). Scale bar, 100 μm.

**
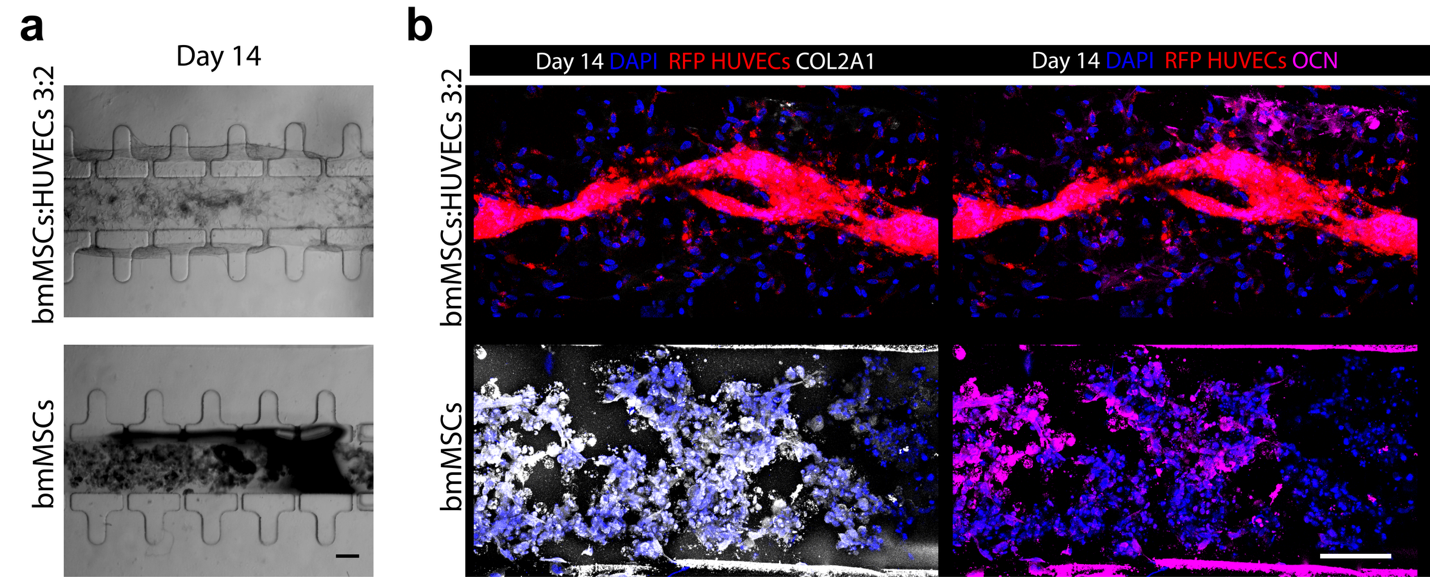
**

**Figure S4. bmMSCs and HUVECs co-cultures at day 14. a.** Representative brightfield pictures of bmMSCs: HUVECs, 3:2 co-cultures and bmMSCs single cultures at day 14. Total cellular density = 50 x 10^6^ cells ml^-1^ (n≥3 independently cultured constructs for each condition). Scale bar, 100 μm. **b.** Representative immunofluorescence images of bmMSCs: HUVECs, 3:2 co-cultures and bmMSCs single cultures at day 14 showing the involution of the vascular networks. Total cellular density = 50 x 10^6^ cells ml^-1^  (n≥3 independently cultured constructs for each condition). Scale bar, 100 μm.


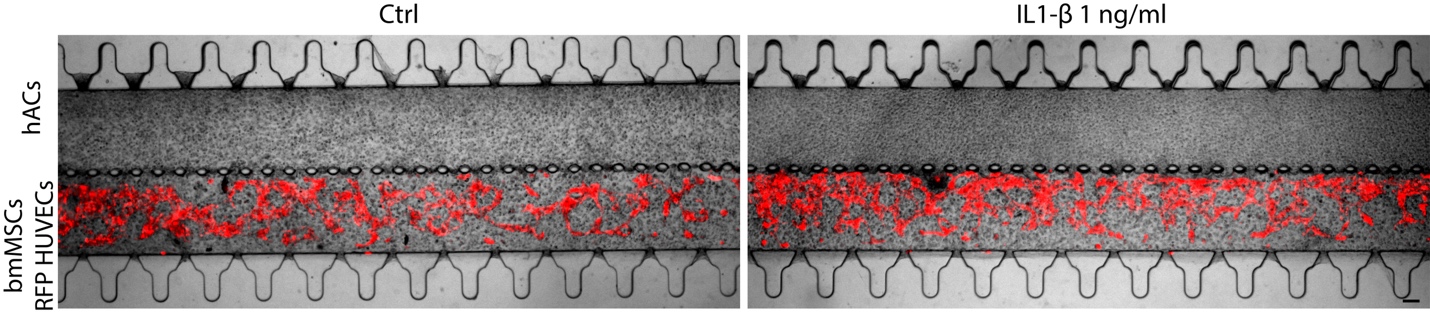


**Figure S5. Overview of vascular structures upon IL-1β administration.** Representative images of a vascularized osteochondral construct at day 7 in controls and construct exposed to 1 ng ml^-1^ of IL-1β (bmMSCs: RFP HUVECs 3:2). Brightfield and fluorescent images are overlaid. RFP^+^ HUVECs vascular networks are represented in red (n = 4 independently cultured samples). Scale bar, 100 µm.

| **Primary human Articular Chondrocytes (hACs)** | **age** | **sex** | **tissue source** | **anatomical location of harvested tissue** |
| --- | --- | --- | --- | --- |
| Donor 1 | 65 | female | cadaver | knee, |
| Donor 2 | 32 | female | cadaver | knee |
| Donor 3 | 54 | male | cadaver | knee |
| Donor 4 | 50 | male | surgical, traumatic | knee |
| Donor 5 | 51 | male | cadaver | knee |
| Donor 6 | 79 | female | cadaver | knee |
| Donor 7 | 47 | male | surgical, traumatic | knee |
| **bone marrow derived Mesenchymal Stromal Cells (bmMSCs)** | **age** | **sex** | **tissue source** | **anatomical location of harvested tissue** |
| Donor 1 | 25 | male | bone marrow aspirate | iliac crest |
| Donor 2 | 17 | male | bone marrow aspirate | iliac crest |
| Donor 3 | 39 | female | bone marrow aspirate | iliac crest |
| Donor 4 | 43 | male | bone marrow aspirate | iliac crest |
| Donor 5 | 47 | female | bone marrow aspirate | iliac crest |

**Table S1: Demographics of hACs and bmMSCs donors used in this study.**
